# Patient safety error reporting program for future undergraduate nursing education: A scoping review protocol

**DOI:** 10.1371/journal.pone.0273737

**Published:** 2022-08-31

**Authors:** Mi Ok Song, So Young Yun, Aeri Jang

**Affiliations:** Department of Nursing, Nambu University, Gwangju, South Korea; University of Oxford, UNITED KINGDOM

## Abstract

Patient safety error reporting education is instrumental in promoting a culture of safety where health-care providers learn from errors and prevent such problems from being repeated. The proposed scoping review aims to establish a comprehensive understanding of how patient error reporting education has been implemented in undergraduate nursing education and present a direction for developing a future patient safety reporting program. The proposed scoping review protocol will be conducted using the Arksey and O’Malley methodological framework, following the Joanna Briggs Institute’s methodology for scoping reviews. It will be reported according to Preferred Reporting Items for Systematic reviews and Meta-Analyses for Protocols (PRISMA-P), and a full scoping review will be reported according to PRISMA extension for Scoping Review (PRISMA-ScR). In the scoping review, studies published in any language and where the participants were undergraduate nursing students were included. In addition, the search period will not be limited, and the following databases will be used to search for relevant studies: MEDLINE, CINAHL, and Excerpta Medica databases (EMBASE). Moreover, this scoping review does not include unpublished studies or gray literature. Two reviewers will independently review titles and abstracts to evaluate inclusion and exclusion criteria, and primary literature will be selected. Two reviewers will independently assess the full text of selected primary literature in detail against the study criteria.

## Introduction

Patient safety is defined as “reducing the risk of preventable, harmless, and unnecessary harm to the minimum acceptable level in the process of providing health care [1].” It is evaluated as a major value in health-care institutions [2], medical accidents, or near-misses of health-care providers that threaten patient safety. Thus, it is necessary to analyze and learn about such errors in academic and clinical settings to improve the quality of medical care [3].

The reporting rate on patient safety error is very low, with a median of 2 in the 1–66 range [4]. Furthermore, improving healthcare providers’ perception and attitude toward patient safety error reporting will significantly influence the decision-making process for reporting [5]. Thus, health-care providers must share information and learn about patient safety errors and establish a safe reporting culture before reorganizing the system for Patient Safety Error Reporting (PSER). Changing the systematic environment, such as policies, structures, and processes, will be essential to forming a safe reporting culture [6]. Therefore, educating undergraduate nursing students who will become key personnel of future health care providers on the patient safety error reporting system will be an important framework for establishing a safety reporting culture.

However, when nursing students are exposed to patient safety-related problem situations, there are insufficient opportunities to directly experience the process of solving problems through appropriate communication and cooperation between medical staff [7]. Furthermore, when nursing students experience errors, they do not report errors out of fear of the consequences or lack of awareness of the situation [8]. Therefore, implementing patient safety error reporting education based on various teaching methods and learning strategies within the undergraduate education for nursing students is required [2, 9]. Such education should be designed so that nursing students can actively participate in changing social systems and individual lifestyles to form a sustainable culture after completing the undergraduate course and moving to clinical practice.

The 2030 United Nations Agenda presents a new and comprehensive goal for sustainability that future societies should aim to achieve, and quality education is presented as one of the core goals [7]. As an educational paradigm to adapt to these future societies, the transition to e-learning is essential. Fortunately, e-learning frameworks for sustainable development have been developed [8], including an e-learning framework for knowledge management [9] and a systematic theoretical framework for e-learning [10]. However, the e-learning framework for achieving sustainable goals in the post-COVID era will differ. Therefore, analyzing the patient safety report education program based on the e-learning framework developed by Zhang et al. [8] is a meaningful challenge for the sustainable development of future nursing education.

A study on nursing students’ patient safety error reporting education program is as follows. Team activities on error reporting increased knowledge of error reporting and interprofessional team performance [11]. Similarly, interprofessional team-based learning activities related to “learning from errors” effectively improved attitudes toward error reporting [12]. The online approach for developing critical incident reporting skills of nursing students was effective in the incident report performance and confidence of nursing students [13]. However, finding a scoping review of these patient safety error reporting educational programs targeting nursing students has been difficult. In literature reviews related to patient safety error reporting, a review was conducted on the content of patient safety in the program that nursing students learn [9, 10] and an overview of medical error in hospitals [14]. However, there were no scoping reviews on any educational programs on patient safety reporting, which are important in raising awareness before accidents occur and providing theoretical knowledge about patient and general safety accidents and patient education contents. Moreover, it was challenging to find a scoping review study on developing an educational program for future undergraduate nursing education.

This scoping review reviewed the literature on education programs about error reporting in the undergraduate nursing curricula. In particular, the proposed scoping review aims to establish a comprehensive understanding of how patient error reporting education has been implemented in undergraduate nursing education and present a direction for developing a future patient safety reporting education program. This review asks the following questions.

1. What features of the patient safety error reporting education program are included in undergraduate nursing education?
2. What are the challenges of using a patient safety error reporting education program for future undergraduate nursing education?

## Materials and methods

This proposed scoping review protocol will be conducted using the Arksey and O’Malley methodological framework [15] and follows the Joanna Briggs Institute’s methodology for scoping reviews [16]. This scoping review protocol was reported according to Preferred Reporting Items for Systematic reviews and Meta-Analyses for Protocols (PRISMA-P) [17], and a full scoping review will be reported according to PRISMA extension for Scoping Review (PRISMA-ScR) [18].

### Inclusion criteria

#### Participants

Studies with undergraduate nursing student participants and those that discuss error report education programs for undergraduate nursing students were included in this review. Studies of error reporting education programs for graduate (magisterial or doctoral) or practicing (licensed vocational nurses, registered nurses, advanced practice nurses, or the equivalent) nurses will be excluded. Similarly, tool development, meta-analyses, and review papers will be excluded.

#### Concept

This study intends to review the research on the PSER education program applied to nursing students in the undergraduate course. Studies that describe strategies to enhance the PSER experience in the program and allow for sustainable development, taking into account clinical relevance, will be included in this review.

#### Context

In this review, where PSER education programs were presented in academic or clinical settings, and studies of virtual simulations, were included. Studies related to scenario development and those about education programs for simple patient safety knowledge transfer in research methodology, or nursing theory courses were not relevant to this review’s scope on patient safety error reporting and were excluded.

#### Types of sources

This scoping review is not limited to design-based studies based. Both experimental and quasi-experimental study designs will be considered. In addition, observational and descriptive studies will be considered for inclusion, and qualitative studies with content related to our review questions. However, opnion or editorial articles that do not describe the design of the program are excluded.

### Methods

#### Search strategy

Relevant published studies were collected, and a limited initial search was performed on MEDLINE and Cumulative Index of Nursing and Allied Health Literature (CINAHL) to identify articles on the subject. Text and index terms from related articles were used to develop an overall search strategy for MEDLINE (Table 1). The search strategy is tailored for each database it contains, and reference lists of all included articles are screened for further study. This review included studies published in different languages, and the search period will not be limited. Databases to search include the MEDLINE and CINAHL databases and Excerpta Medica (EMBASE). Unpublished studies or gray literature are not included in this scoping review.

**Table 1:** Search strategy for MEDLINE (PubMed).

No.	Query	Results retrieved
1	medical error [Mesh]	118,366
2	Safety [Mesh]	85,857
3	risk management [Mesh]	330,998
4	medical error [Title/Abstract] OR medical mistake*[Title/Abstract] OR, medical error*[Title/Abstract] OR surgical error*[Title/Abstract] OR critical incident*[Title/Abstract] OR medical incident*[Title/Abstract] OR never event*[Title/Abstract] OR near miss*[Title/Abstract] OR unexpected harm*[Title/Abstract] OR clinical incident*[Title/Abstract] OR harmful incident*[Title/Abstract] OR patient safety incident*[Title/Abstract]	11,928
5	#1 OR #2 OR #3 OR #4	501,784
6	truth disclosure [Mesh]	15,264
7	reporting [Title/Abstract]	218,350
8	risk report*[Title/Abstract] OR incident report*[Title/Abstract] OR error report*[Title/Abstract] OR safety report*[Title/Abstract] OR reporting system [Title/Abstract] OR learning system [Title/Abstract] OR adverse event report*[Title/Abstract] OR voluntary report*[Title/Abstract] OR mandatory report*[Title/Abstract]	15,707
9	#6 OR #7 OR #8	236,407
10	nursing students [Mesh]	27,349
11	baccalaureate nursing education [Mesh]	19,657
12	student*[Title/Abstract] OR “nursing students” [Title/Abstract] OR “student nurses” [Title/Abstract]	314,282
13	training[Title/Abstract] OR teaching[Title/Abstract] OR education[Title/Abstract] OR nursing education[Title/Abstract] OR “nursing curriculum”[Title/Abstract] OR “nursing curricula”[Title/Abstract] OR “nursing knowledge”[Title/Abstract] OR “teaching methodologies”[Title/Abstract] OR “nursing programs”[Title/Abstract] OR “teaching content”[Title/Abstract] OR “undergraduate nursing”[Title/Abstract] OR “undergraduate nursing curriculum”[Title/Abstract] OR “undergraduate nursing curricula”[Title/Abstract]	1,025,642
14	#10 OR #11 OR #12 OR #13	1,034,470
15	#5 AND #9 AND #14	189

#### Study/Source of evidence selection

After data collection, all citations identified will be collated and uploaded to EndNote X8 8.2 (Clarivate Analytics, Philadelphia, PA, USA), and duplicates will be removed. Two reviewers will independently review titles and abstracts to evaluate inclusion and exclusion criteria, and primary literature will be selected. The full text of selected primary literature will be independently evaluated by two reviewers in detail against the study criteria. The reasons for excluding full-text articles are reported in the scoping review. During the selection process, any disagreements between the judges will be resolved through discussion. The search and study inclusion process results will be reported in full in the final scoping review and presented in the PRISMA-ScR Flowchart [17].

#### Data extraction

Data will be extracted by one reviewer using a data-extraction tool developed by the reviewers (All Authors). They will be verified by another reviewer. The general characteristics of the selected articles in this review will be extracted based on the Joana Briggs Institue’s scoping review data extraction section [19]. The features of the PSER program will be extracted, including contents and teaching strategies and outcome level. In particular, contents will be extracted based on the incident type suggested by WHO in 2009 [20], and outcome levels will be extracted based on Kirkpatrick’s [21] four levels of evaluating training programs. The future PSER nursing education challenge will be extracted based on an e-learning framework [8], and the details of the extraction tool are presented in Table 2. A pilot review will be performed with the first two included studies in this scoping review.

**Table 2:** Extraction tool.

Categories	Sub-categories	Details
General characteristics	Authors	
	Year of publication	
	Country of origin	
	Sampling	
Review question 1: Features	Incident type	Error, near miss, adverse event, sentinel event
	Teaching strategies	
	Outcomes level	React, learning, behavior, outcome
Review question 2: Challenges	Model for sustainable development education	Level A (Identifying macro and micro problems), Level B (Studying causes and preconditions behind these problems), Level C (Developing hard skills), Level D (Developing verbal speech skills), Level E (Developing organizational skills), Level F (Developing soft skills and professional vision), Level G (Developing innovative thinking)

The data-extraction tool can be modified and revised as necessary during data extraction. The modifications will be described in the final scoping review. Any disagreements that arise between the reviewers in the data extraction process will be resolved through discussion.

#### Data analysis and synthesis

A full-scale analysis of the selected literature will be performed according to the review questions and the results will be tabulated according to the data extraction tool. This section will include a summary that logically explains the results of the research questions. The results of the challenge for future nursing education will be presented through narrative synthesis.

### Data presentation

The study results will be provided in a table according to the key factors of the model for sustainable development education. Quantitative data will use descriptive statistics, and narrative summaries and tabulated results will be used to characterize patient safety incident reporting programs. Because of this, the results of this study will reveal strategies for the sustainable development of students’ patient safety error reporting experience process.

### Ethical consideration

This study will begin analysis after receiving approval of the application for exemption from deliberation by the Institutional Review Board of Nambu University (IRB #1041478-2021-HR-035).

## Discussion

Identifying causes and finding and improving system vulnerabilities are crucial to preventing patient safety accidents [22]. These aspects are necessary to prevent similar incidents by determining what happened and how and why the incident occurred. In Korea, the Patient Safety Reporting Learning System noted 563 safety accidents in 2016 and more than 1,000 safety accidents every month in 2019. However, it is difficult to determine the exact type and distribution of patient safety accidents because hospitals are reluctant to disclose them [23]. In addition, because patient safety accident reporting is left to the health-care provider’s discretion, it is expected that there will be more cases in reality.

Nurses account for the highest proportion of health-care providers. As a workforce dedicated to patient safety, they educate and collaborate with other medical staff to prevent patient safety incidents and play a key role in error reporting. Therefore, this study will analyze the nurse educator’s strategy for more effective program development by reviewing the patient safety error reporting education program for undergraduate nursing students who are future prospective nurses. Based on the data extracted according to the model for sustainable development education [8] programs, the current situation will be reflected on. Then, the optimal patient safety error reporting education program design and nursing educator’s strategy that can be connected with future education will be discussed. Any changes to the protocol’s content during the scoping review will be detailed, and the limitations of the review will be described. All these changes will be shared by Open Science Framework (OSF registration DOI: 10.17605/OSF.IO/G9BTX).

## Conclusions

This review will identify the current status of the developed PSER education program and suggest a challenging direction for the program for undergraduate nursing education in the future. It will be used as primary data for developing educational programs for PSER in nursing education. Likewise, the global community can be used as primary data for one educational method to achieve the core goal of “quality education” as part of the 2030 United Nations Agenda, a sustainable goal that future societies should aim to achieve.

## Supporting information

S1 Checklist Reporting checklist for protocol of a systematic review and meta analysis. (DOCX)
